# Metabolite diversity among representatives of divergent *Prochlorococcus* ecotypes

**DOI:** 10.1128/msystems.01261-22

**Published:** 2023-10-10

**Authors:** Elizabeth B. Kujawinski, Rogier Braakman, Krista Longnecker, Jamie W. Becker, Sallie W. Chisholm, Keven Dooley, Melissa C. Kido Soule, Gretchen J. Swarr, Kathryn Halloran

**Affiliations:** 1 Department of Marine Chemistry and Geochemistry, Woods Hole Oceanographic Institution, Woods Hole, Massachusetts, USA; 2 Department of Earth, Atmospheric, and Planetary Sciences, Massachusetts Institute of Technology, Cambridge, Massachusetts, USA; 3 Department of Civil and Environmental Engineering, Massachusetts Institute of Technology, Cambridge, Massachusetts, USA; 4 Science Department, Alvernia University, Reading, Pennsylvania, USA; 5 Department of Biology, Massachusetts Institute of Technology, Cambridge, Massachusetts, USA; 6 Department of Ecology and Evolution, University of Chicago, Chicago, Illinois, USA; 7 MIT/WHOI Joint Program in Oceanography/Applied Ocean Sciences and Engineering, Department of Marine Chemistry & Geochemistry, Woods Hole Oceanographic Institution, Woods Hole, Massachusetts, USA; University of California San Diego, La Jolla, California, USA

**Keywords:** *Prochlorococcus*, metabolomics, DNA methylation

## Abstract

The euphotic zone of the surface ocean contains distinct physical-chemical regimes that vary in light and nutrient concentrations as an inverse function of depth. The most numerous phytoplankter of the mid- and low-latitude ocean is the picocyanobacterium *Prochlorococcus*, which consists of ecologically distinct subpopulations (i.e., “ecotypes”). Ecotypes have different temperature, light, and nutrient optima and display distinct relative abundances along gradients of these niche dimensions. As a primary producer, *Prochlorococcus* fixes and releases organic carbon to neighboring microbes as part of the microbial loop. However, little is known about the specific molecules *Prochlorococcus* accumulates and releases or how these processes vary among its ecotypes. Here, we characterize the metabolite diversity of *Prochlorococcus* by profiling three ecologically distinct cultured strains: MIT9301, representing a high-light-adapted ecotype dominating shallow tropical and sub-tropical waters; MIT0801, representing a low-light-adapted ecotype found throughout the euphotic zone; and MIT9313, representing a low-light-adapted ecotype relatively most abundant at the base of the euphotic zone. In both intracellular and extracellular metabolite profiles, we observe striking differences across strains in the accumulation and release of molecules, such as the DNA methylating agent S-adenosyl-methionine (intracellular) and the branched-chain amino acids (intracellular) and their precursors (extracellular). While some differences reflect variable genome content across the strains, others likely reflect variable regulation of conserved pathways. In the extracellular profiles, we identify molecules such as pantothenic acid and aromatic amino acids that may serve as currencies in *Prochlorococcus*’ interactions with neighboring microbes and, therefore, merit further investigation.

**IMPORTANCE:**

Approximately half of the annual carbon fixation on Earth occurs in the surface ocean through the photosynthetic activities of phytoplankton such as the ubiquitous picocyanobacterium *Prochlorococcus*. Ecologically distinct subpopulations (or ecotypes) of *Prochlorococcus* are central conduits of organic substrates into the ocean microbiome, thus playing important roles in surface ocean production. We measured the chemical profile of three cultured ecotype strains, observing striking differences among them that have implications for the likely chemical impact of *Prochlorococcus* subpopulations on their surroundings in the wild. Subpopulations differ in abundance along gradients of temperature, light, and nutrient concentrations, suggesting that these chemical differences could affect carbon cycling in different ocean strata and should be considered in models of *Prochlorococcus* physiology and marine carbon dynamics.

## INTRODUCTION

*Prochlorococcus* is the most numerous Cyanobacteria lineage in the oceans and fixes ca. 4 Gt of carbon annually (1), contributing approximately 10% of oceanic carbon fixation. Primary producers such as *Prochlorococcus* anchor the surface ocean microbiome, a consortium of bacteria, phytoplankton, archaea, viruses, and grazers that fix and recycle carbon. Interactions within the microbiome rely on the chemical exchange of metabolites among organisms, facilitating the turnover of 25 Gt of carbon per year (2). Despite their centrality in the carbon cycle, the identities of most of these metabolites remain elusive, with few constraints on their sources (e.g., *Prochlorococcus*), sinks (e.g., mixotrophic or heterotrophic bacteria), and/or the growth and genetic factors that affect metabolite release and composition (3).

One challenge for predicting metabolite production in *Prochlorococcus* is that this genus harbors vast genetic diversity, with unknown impacts on metabolite composition. At the highest level, major branches of the *Prochlorococcus* tree form ecologically coherent populations (or “ecotypes”) whose relative abundances shift along large-scale gradients of light, temperature, and nutrients (4 – 8). A central axis of ecological differentiation is seen most clearly in warm stable water columns: recently diverging high-light (HL)-adapted ecotypes dominate near the surface, where light is abundant and nutrient levels are low or undetectable, while deeply branching low-light (LL)-adapted ecotypes increase in abundance deeper in the water column where they encounter low light and elevated nutrients (9 – 11). Additional niche differentiation within *Prochlorococcus* arises due to variations in temperature as a function of latitude (7) and variations in nutrient concentrations in different ocean regions (12 – 14). While the evolutionary diversification of ecotypes has been linked to genetically encoded changes in some core metabolic pathways (15), evolutionary changes in other pathways are not yet systematically understood.

These broadly defined ecotypes further display significant genomic diversity within coexisting sub-clades (16, 17). Due to this microdiversity, *Prochlorococcus* has a very large pangenome (i.e., the collective genome of all cells) despite individual cells harboring highly streamlined genomes that lack canonical horizontal gene transfer mechanisms and instead appear to exploit novel mobile genetic elements called tycheposons (18). Approximately 1,000 genes represent the shared core genome (19), with the pangenome of the *Prochlorococcus* “collective” projected to be over 80,000 genes (9). The genetic diversity in the pangenome enables differential responses by cells to changes in nutrient availability including phosphorus (20), nitrogen (21, 22), and iron (23, 24). As the level of genetic identity between *Prochlorococcus* strains increases, pairwise analysis of genomes reveals decreases in the number of unique genes (9). In this study, we focused our interpretations on metabolites within central carbon pathways which are generally conserved (with a few exceptions noted below) within the core genome. In summary, the hierarchical patterns of diversity reflected in its pangenome have allowed *Prochlorococcus* to achieve a global distribution, high abundance, and ecological stability in the face of myriad and variable environmental forces in the euphotic zone across the world’s oceans (9).

*Prochlorococcus* plays an important role in the global carbon cycle through fixation and subsequent release of organic carbon. However, the composition of the released material, particularly labile molecules such as metabolites, has not been studied systematically, nor have different strains representing diverse ecotypes been compared in this regard. Intracellular metabolites participate in biochemical reactions that power the cell, build biomass, and enable responses to stressors in a changing environment, while extracellular metabolites leave the cell through active and passive release of biochemical by-products, signaling molecules, and waste materials (reviewed in reference 3). In practice, intracellular metabolites reflect near-time response and, therefore, an instantaneous phenotype, while extracellular metabolites reflect a time-integrated metabolic phenotype (at least in batch culture). Large-scale genomic differences among strains or organisms under study result in distinctive metabolite profiles in both intra- and extracellular metabolite pools (25, 26). Indeed, metagenomic data from microbial consortia have been used to infer metabolite composition differences within consortia members (27 – 29). At the strain level, parallel work with human-derived bacteria showed differences in intra- and extracellular metabolite profiles (30 – 32). In short, variations in metabolite concentrations and composition are expected with genomic variability, but they could also arise from distinct responses to environmental stressors and/or differences in gene regulation and output. The degree to which genomic variability within *Prochlorococcus* ecotypes shapes metabolite composition is currently unknown.

As a first step in understanding metabolite production by *Prochlorococcus*, we chose three strains from phylogenetically and physiologically divergent ecotypes for growth under nutrient-replete conditions. MIT9301 is a member of the recently diverged HLII ecotype, which dominates the tropics and sub-tropics at the shallowest depths and is the most abundant *Prochlorococcus* ecotype globally (10). MIT0801 is a member of the LLI ecotype, which is often abundant throughout the euphotic zone with maxima at middle depths, typically 50–100 m (33). MIT9313 is a member of the deeply branching LLIV ecotype whose abundances increase with depth, typically reaching its highest abundance below 100 m (10). The LLI ecotype can tolerate light fluctuations associated with periods of deep mixing during the winter months, when they often become relatively abundant (10, 34). Using mass-spectrometry-based metabolomics, we ask (i) how the intra- and extracellular metabolite composition varies among genetically divergent ecotypes; (ii) how closely patterns in intracellular metabolites reflect genetic variability; and (iii) which extracellular metabolites are released at sufficiently high concentrations to be candidates for facilitating interactions with other microbes. Due to the known variability in growth rates with light levels for these ecotypes (5, 8), we collected intracellular and extracellular metabolite profiles at the same light level and at light levels closer to anticipated field conditions (see Materials and Methods). We separated intracellular metabolites and extracellular metabolites by filtration, such that intracellular metabolites represent those present within *Prochlorococcus* biomass caught on 0.1-µm filters and the extracellular metabolites represent those released into the spent growth media.

## RESULTS AND DISCUSSION

Because the close relatives of our chosen strains generally thrive in different environmental regimes in the wild (10), we considered our metabolite profiles in the context of metabolic processes influenced by key variables in those environments such as light intensity and nutrient availability. This approach focused our analysis on groups of metabolites rather than on individual metabolites within disparate metabolic pathways (as might result from a ranked-order approach) that may be affected more by analytical precision than by biological variability (35).

**Fig 1:**
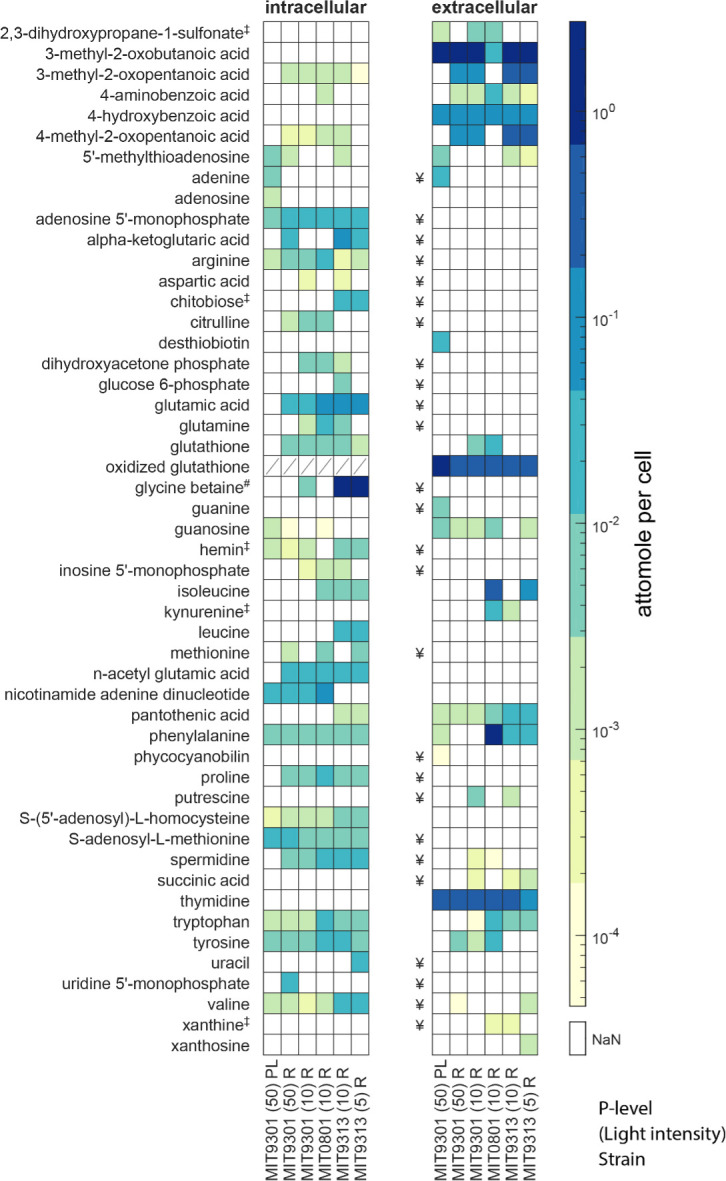
Metabolites detected in three strains of *Prochlorococcus* within intracellular and extracellular phases. Metabolites are presented in alphabetical order with concentrations in attomoles per cell; ‡ indicates metabolites whose biosynthesis genes were not found in the genome, and # indicates a metabolite whose biosynthesis genes are present only in MIT9313. Extracellular metabolites with ¥ have an extraction efficiency below 1% and cannot be reliably quantified in the method used here. Intracellular concentrations of oxidized glutathione are not available due to interference by an unknown compound. Columns refer to MIT9301 (HLII) light intensity 50 µmol photons m^−2^ s^−1^, MIT9301 light intensity 10 µmol photons m^−2^ s^−1^, MIT0801 (LLI) light intensity 10 µmol photons m^−2^ s^−1^, MIT9313 (LLIV) light intensity 10 µmol photons m^−2^ s^−1^, and MIT9313 light intensity 5 µmol photons m^−2^ s^−1^. PL are the phosphate-limited cultures; R is phosphate replete.

We examined the role of light by growing all three strains at the same light level and by growing two strains at the light level that more closely approximated their realized optimum in the wild (growth rates provided in Table S1). We tested nutrient limitation by growing MIT9301 under low-phosphorus and nutrient-replete conditions. Across our six cultures combining treatments and strains, 35 intracellular compounds and 18 extracellular compounds (plus four extracellular metabolites that could not be reliably quantified; Fig. 1) passed our quality control criteria for detection and quantification. These metabolites represent a subset of conserved metabolic processes of interest in adaptations to light and nutrients. We examined intracellular and extracellular metabolite profiles from a pathway perspective in order to consider the dynamics of multiple metabolites at once. We organize these observations and conclusions according to possible driving factors.

### Metabolite changes linked to differences in light levels and nutrient concentrations

**Fig 2:**
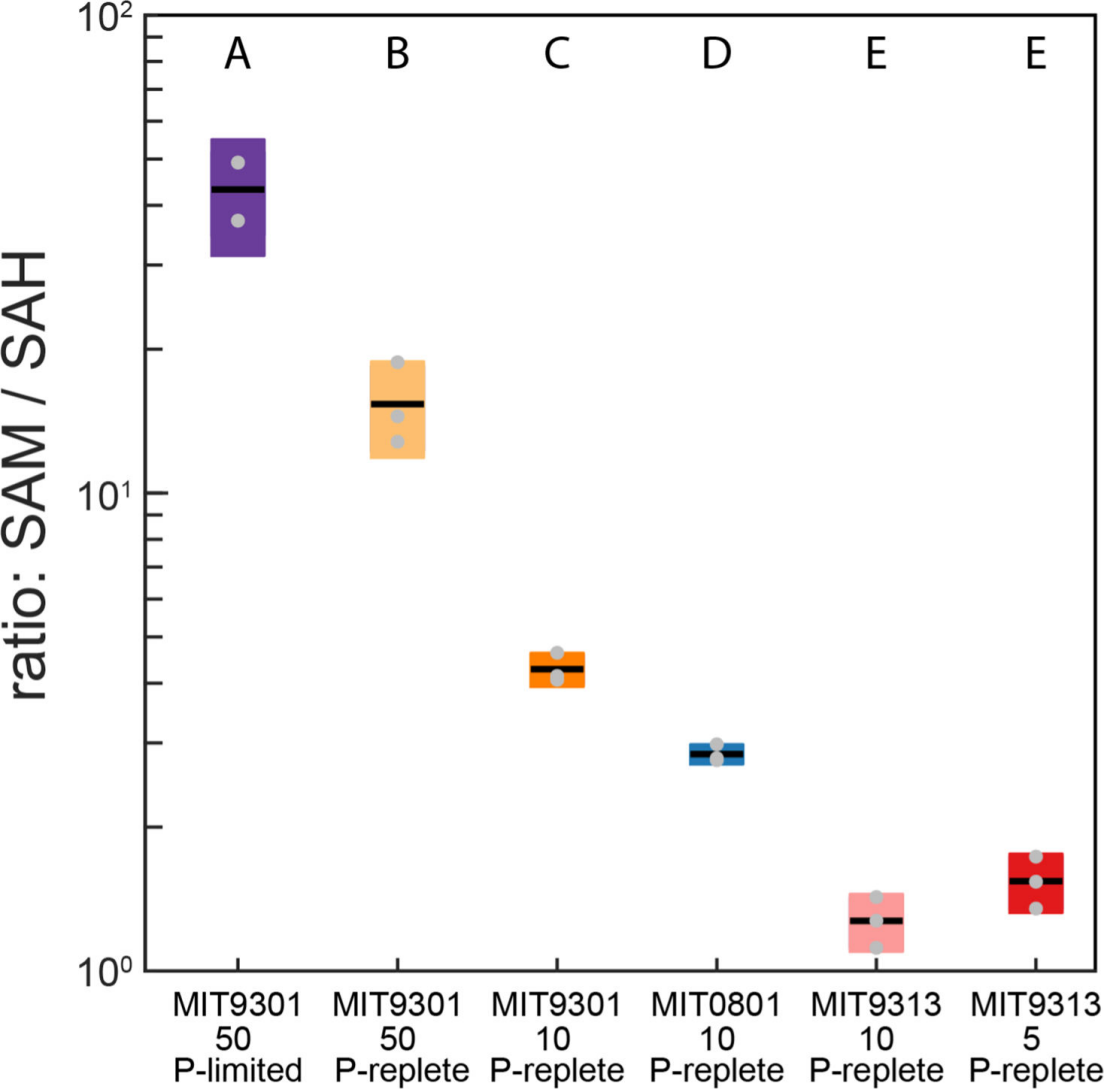
S-adenosyl methionine (SAM) to S-adenosyl-homocysteine (SAH) ratios in three strains of *Prochlorococcus* under different culture conditions. The light intensities used in this study are in Table 1 and are 5, 10, or 50 µmol photons m^−2^ s^−1^. The box-plots represent replicate cultures within each treatment; gray dots indicate individual measurements. Letters above each treatment indicate statistically significant differences (two-way analysis of variance followed by post-hoc test using Fisher’s least significant difference; *P*-value <<0.0001).

A key parameter that varies among the strains (and ecotypes) is the optimal light intensity for growth and irradiances below which they cannot survive. MIT9301 (an HLII strain) is better adapted to high light in the surface ocean, displaying optimal growth rates at light intensities that are inhibitory to low-light-adapted strains. MIT0801 (LLI) and MIT9313 (LLIV), on the other hand, can survive at light levels that will not sustain high-light-adapted ecotypes and have optimal growth rates at light intensities that are limiting to high-light-adapted strains (5, 8). We explicitly considered light exposure in our experimental design, by growing all strains at an identical intermediate light level (10 µmol photons m^−2^ s^−1^) and by growing two strains at light levels (MIT9301, 50 µmol photons m^−2^ s^−1^; MIT9313, 5 µmol photons m^−2^ s^−1^) closer to those where populations in the wild reach their highest densities (Table 1). In each of these two strains, light levels had a minor influence on metabolite concentrations (Fig. 1). Although we see minimal intra-strain differences in metabolite production as a function of light level, we observed clear differences in metabolite levels between strains, including those involved in metabolic processes that could be linked to adaptations or acclimations of individual ecotypes to different light and nutrient conditions in the wild (Fig. 1 and 2).

**TABLE 1:** Light intensities used to cultivate the strains in this study

Strains used in this study	Ecotype	Range of light intensities for ecotype’s maximum growth rate in culture^*a*^ ^,^ ^*b*^ (µmol photons m^−2^ s^−1^)	Light intensities in this study (µmol photons m^−2^ s^−1^)	Light intensity at ecotype’s maximum population density in field studies^*a*^ ^,^ ^*c*^ (µmol photons m^−2^ s^−1^)
MIT9301	HLII	40–150	10 and 50	25–85
MIT0801	LLI	10–50	10	2–6.5
MIT9313	LLIV	10–50	5 and 10	0.5–1.5

^*a*^ To arrive at these optimal light levels in our study, we examined data from previous laboratory studies conducted under diel light:dark conditions typical of what the cells experience in the oceans. We calculated the total photon fluxes experienced during the light period of diel light:dark cycles in those studies and adjusted light intensities so the cells experienced the equivalent photon flux spread over 24 h.

^*b*^ Estimated ranges for the light levels at which growth rates of different ecotypes are maximized in laboratory settings. Not all strains used in this study have been individually characterized, so these ranges reflect the qualitative averages across strains of different ecotypes, as determined in Moore et al. (5).

^*c*^ Estimated (constant light-equivalent) light level for the water column depth at which populations of different ecotypes reach their maximum density in stratified waters during summer months at the Hawaii Ocean Time-series (HOT) and the Bermuda Atlantic Time-series Study site (BATS), as determined from Malmstrom et al. (10).

Two such intracellular metabolites are S-adenosyl-methionine (SAM) and its demethylated product S-adenosyl-homocysteine (SAH), often reported as the ratio of SAM to SAH to highlight the reaction that transfers a methyl group from SAM to other biomolecules, generating SAH. This reaction is a major pathway for methylating DNA, RNA, and proteins, a process that often increases under stress (36). Intracellular concentrations of SAM and SAH are tightly regulated to control the extent of methylation of nucleic acids; thus, many studies have inferred that higher SAM:SAH values reflect increased methylation potential (37, 38). In this study, we calculated SAM:SAH values for all treatments and strains (Fig. 2). We asked two questions in interpreting these data: (i) whether different strains maintained different SAM:SAH values under the same growth conditions and (ii) whether changes in light levels or nutrient concentrations affected SAM:SAH values in a subset of strains.

For the first question, the SAM:SAH values across the three strains grown at the same light level (10 µmol photons m^−2^ s^−1^) are statistically significantly higher for MIT9301 than for either MIT0801 or MIT9313 (Fig. 2). To ascertain whether there was a genomic basis for this observation, we searched the three strain genomes (in Kyoto Encyclopedia of Genes and Genomes [KEGG]) for genes involved in SAM metabolism. The three strains share several enzymes (SAM synthetase [*metK*]; SAM:tRNA isomerase [*queA*], and SAM decarboxylase [*speD*]), but only MIT9301 encodes the gene for a SAM-dependent cytosine methyltransferase (EC.2.1.1.199). Coe et al. (39) recently showed that within *Prochlorococcus,* only strains from the HLII ecotype methylate cytosine residues in their DNA, although MIT9301 was not tested explicitly. Previous work in *Arabidopsis* showed dynamic and widespread methylation of cytosine residues as a response to different stress types (40). Together these observations suggest that the elevated SAM:SAH values in MIT9301 compared to the other strains could reflect a distinct ability of the HLII ecotype to methylate cytosine residues as a stress response. Although we did not explicitly test the impact of stress in this experiment, we can approximate that response by comparing the SAM:SAH values in the three MIT9301 treatments that varied light and P availability (Fig. 2). Here, we note an increase first with higher light and then a further increase with P-limitation (see growth curves in Fig. S1). The latter observation is consistent with a >10-fold increase in extracellular oxidized glutathione, a product of peroxide detoxification, in MIT9301 under P-limited relative to nutrient replete conditions. Because our study did not focus on methylation of nucleosides as a stress response, more work will be needed to explore the mechanisms behind these observations.

**Fig 3:**
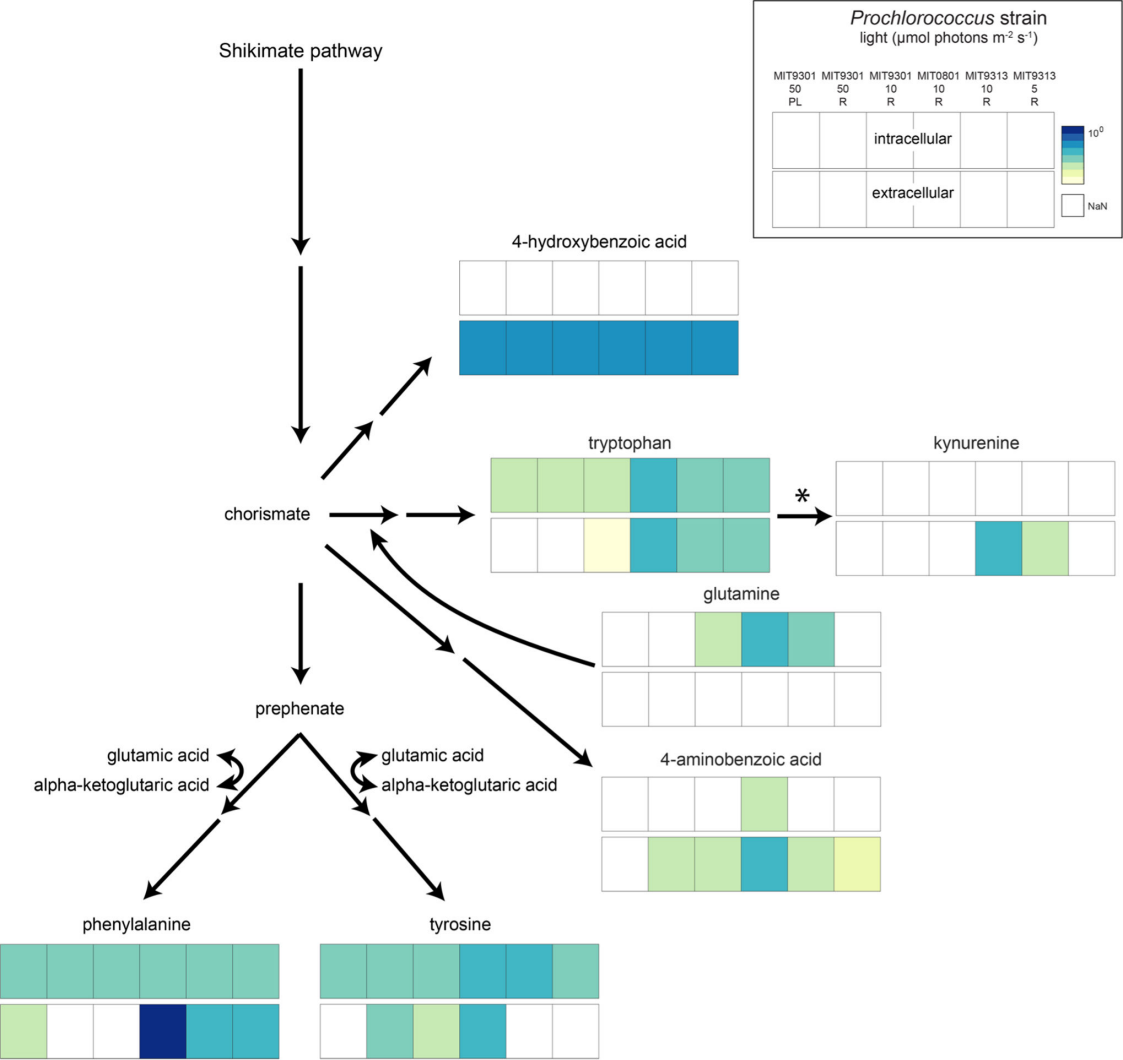
Aromatic amino acids pathway. Squares are log values of mean from Fig. 1 (top bar for each compound, intracellular; bottom bar, extracellular). Light intensities are shown as well as whether the cultures were P-limited (PL) or replete (**R**). A bar graph representation of these data is provided in Fig. S2.

One of the extracellular metabolites that was elevated in MIT9313 under relatively high light was kynurenine, an oxidation product of the aromatic amino acid tryptophan (Fig. 1 and 3). Kynurenine was also excreted by MIT0801 (at higher levels than MIT9313). In plant cells, tryptophan residues in photosystem II proteins can be oxidized to kynurenine by reactive oxygen by-products of photosynthesis (41). During protein remodeling, kynurenine is replaced with tryptophan and excreted or further degraded. The *Prochlorococcus* pangenome does not contain the pathway for kynurenine production or its downstream degradation, so kynurenine excretion in this study (Fig. 1 and 3) suggests that this molecule could be an oxidation product of reactive oxygen species (ROS) in the photosystem proteins, similar to hypotheses raised in previous work with *Synechococcus* (42).

As we considered the kynurenine and SAM:SAH dynamics across our study, we asked whether there might be differences in the aromatic amino acids and their derivatives as *Prochlorococcus* adjusts to different light regimes. We chose these aromatic compounds because aromatic moieties (i.e., the phenyl groups) are highly reactive to light and ROS. The phenyl group’s planar π system can absorb photons and react with oxygen radicals (43, 44). We observed surprisingly different aromatic amino acid excretion patterns among the strains, despite all three strains using the conserved shikimate pathway for aromatic amino acid synthesis (Fig. 3; Fig. S2). The first reaction in the pathway is catalyzed by 3-deoxy-7-phosphoheptulonate synthase (DAHP synthetase), which is allosterically inhibited by either phenylalanine or tyrosine (45). Mutations in other cyanobacteria that eliminated allosteric regulation by one of these compounds led to excretion of the other (45). MIT0801 released both tyrosine and phenylalanine; MIT9313 released only phenylalanine and MIT9301 released only tyrosine, indicating possible strain- or ecotype-level modifications of these metabolic pathways. However, P-limited MIT9301, for which we observed an elevated SAM:SAH value (Fig. 2) and elevated extracellular oxidized glutathione (Fig. 1), released phenylalanine instead of tyrosine. Phenolic compounds are known antioxidants, and phenylalanine is the starting point of the phenylpropanoid pathway that is broadly involved in stress responses in plants (44). Together, these observations suggest that phenylalanine production and excretion are potential stress response mechanisms in *Prochlorococcus*, which merits further study.

A putative link between cellular stress and phenylalanine exudation in *Prochlorococcus* provides context to some unique exudation patterns in *Prochlorococcus* MIT0801, that is, phenylalanine release was 30-fold higher in MIT0801 (1.2 amol cell^−1^) than in the other strains, with phenylalanine in MIT0801 being by far the most abundant extracellular aromatic amino acid in our experiment. Furthermore, extracellular 4-hydroxybenzoic acid, another phenolic compound emerging from the shikimate pathway, is higher in MIT0801 than in the other strains and is also enhanced in MIT9301 under P-limited relative to nutrient-replete conditions (Fig. 3; Fig. S2). However, extracellular oxidized glutathione, the by-product of peroxide detoxification that is greatly enhanced in MIT9301 under P-limited conditions, is at similar levels in MIT0801 as in other strains (Fig. 1). Thus, exudation patterns of MIT0801 share some similarity to *Prochlorococcus* cells under stress, but the cells do not appear to have been actively experiencing stress in our experiment. Compared to other ecotypes, the LLI ecotype, to which MIT0801 belongs, is highly adapted to periods of deep winter mixing associated with rapidly fluctuating environmental conditions and has a greatly enhanced repertoire of “high light inducible” (*hli*) proteins, a diverse class of genes involved in general stress responses (19). Perhaps, the patterns of metabolite exudation observed in MIT0801 reflect a kind of constitutively “stressed” state that is part of the unique adaptations of the LLI clade to conditions of rapid environmental variation. Further work with different light levels and nutrient concentrations in MIT0801, as well as comparisons to other strains, could help determine how the shikimate pathway is regulated in *Prochlorococcus* and under what conditions.

### Metabolite changes linked to changes in nitrogen acquisition and recycling pathways

The ecotypes in our study experience different nutrient concentrations in the wild, with high-light-adapted strains typically experiencing lower nitrogen and phosphorus concentrations than the low-light-adapted strains. We explicitly tested phosphorus limitation for MIT9301 in this study but kept nitrogen (ammonium) concentrations equivalent among all experiments. We were, therefore, intrigued to observe higher cell-normalized levels of intracellular amino acids in the LL strains, MIT9313 and MIT0801, than in the HLII strain MIT9301 (Fig. 1; Fig. S2). The variability in cell-normalized amino acid concentrations (Fig. S2) was notable given that nearly all amino acid biosynthesis pathways are part of the conserved core genome of *Prochlorococcus* (19). We wondered whether the variability in amino acid levels could reflect the known niche partitioning of *Prochlorococcus* ecotypes along an axis from low nitrogen in the surface ocean (the HL ecotypes) to higher nitrogen deeper in the euphotic zone (the LL ecotypes), specifically in the metabolic processes responsible for nitrogen assimilation, recycling, and storage.

**Fig 4:**
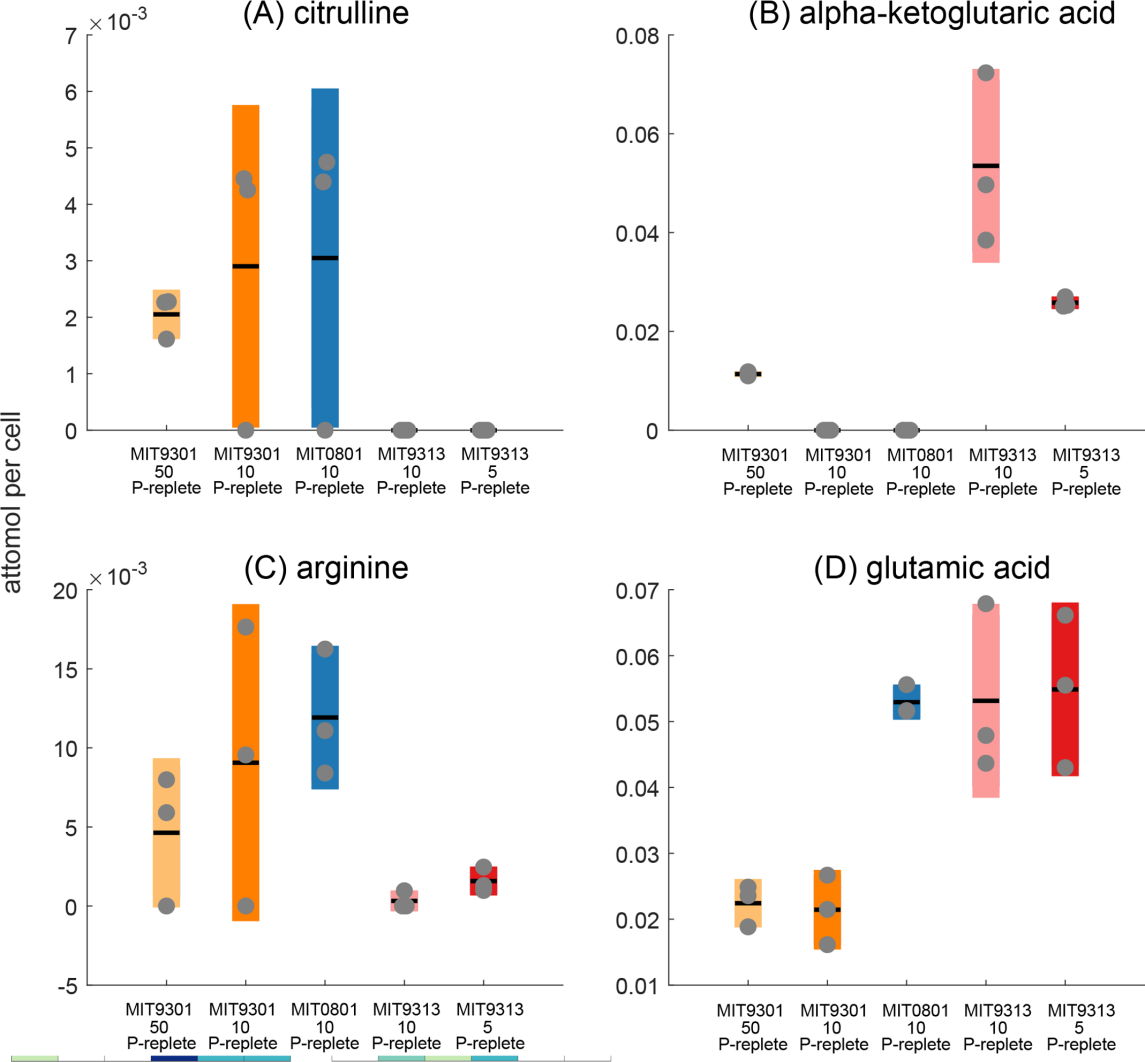
Intracellular cell-specific concentrations of metabolites associated with the urea cycle (**A **and **C**), compared to metabolites associated with inorganic nitrogen acquisition (**B** and **D**). Box plots show mean concentrations and standard deviation of experimental triplicates, with gray dots representing individual samples (orange, MIT9301; blue, MIT0801; red, MIT9313).

To address this question, we examined four metabolites that represent differences in the ecotypes’ ability to recycle organic nitrogen and in the properties associated with the two pathways for ammonium assimilation. Citrulline and arginine are two molecules associated with the urea cycle, a common nitrogen recycling strategy (46), while alpha-ketoglutaric acid and glutamic acid are central metabolites involved in assimilating inorganic nitrogen into protein and nucleic acids (Fig. 4). We focused on intracellular data from the strains grown under replete conditions to avoid complications associated with altered metabolism under P-limited growth. Citrulline and arginine were positively correlated with one another (*R* = 0.83; *P* = 0.001), while alpha-ketoglutaric acid was negatively correlated with citrulline and arginine (*R* = −0.62 and −0.64, respectively; *P* = 0.03 for both). We observed the highest intracellular concentrations of citrulline and arginine in MIT9301 (HLII) and MIT0801 (LLI) and undetectable or low concentrations in MIT9313 (LLIV). In contrast, MIT9313 accumulated high intracellular concentrations of alpha-ketoglutaric acid and glutamic acid. The increased accumulation of citrulline and arginine, relative to alpha-ketoglutaric acid and glutamic acid, in the high light strain (MIT9301) may indicate a greater tendency to use aspects of the urea cycle by this ecotype, even though *Prochlorococcus* cannot perform the canonical urea cycle due to the absence of the gene for arginase production. Previous field and laboratory work has shown that *Prochlorococcus* uses urea and other forms of organic nitrogen in nutrient-depleted environments, although differences among ecotypes have not been systematically explored (4, 47 – 50). Further work with nitrogen-limited cultures may provide additional insights into N cycling under these conditions.

To make sense of the alpha-ketoglutaric acid and glutamic acid concentrations in our study, we considered the two pathways for inorganic nitrogen assimilation: (i) the glutamine synthase (GS)-glutamine oxoglutarate aminotransferase (GOGAT) pathway which converts glutamic acid to glutamine and then combines glutamine with alpha-ketoglutaric acid to produce two glutamic acid molecules and (ii) the glutamic acid dehydrogenase (GDH) pathway which combines alpha-ketoglutaric acid and ammonium to produce glutamic acid and can work in the reverse direction for organic nitrogen recycling. The GDH pathway is considered a poorer route for glutamic acid biosynthesis because this enzyme has a lower affinity for ammonium, relative to GS. However, the GS-GOGAT pathway consumes ATP, while the GDH pathway does not. All *Prochlorococcus* strains encode the high-affinity endergonic GS-GOGAT pathway, but of the strains tested here, only MIT9313 retains the low-affinity but more energetically efficient GDH pathway (19, 51). Flux balance models have shown that low-energy-consuming reactions such as the GDH pathway are favored for ecotypes like MIT9313 at the base of the euphotic zone (52).

Both the GS-GOGAT and GDH pathways have been implicated in responses to N stress in transcriptomics, proteomics, and metabolomics studies in *Prochlorococcus* (21, 51, 53 – 55). Taken together, published studies imply that nitrogen stress induces dynamic responses in transcripts, proteins, and metabolites within the GS-GOGAT pathway for high-light-adapted ecotypes, with lower-amplitude responses in these measurements for low-light ecotypes. For low-light ecotypes, the GDH pathway is not differentially expressed under N stress and could be important for recycling nitrogen from amino acids (51). In this study, all our strains were grown under N-replete conditions, yet we observed elevated alpha-ketoglutaric acid concentrations (Fig. 4) in MIT9313 (LLIV), in contrast to MIT9301 (HLII) and MIT0801 (LLI) and previously published results for VOL29 (HLI) (55). Although transcriptomics data are required to confirm this result, it suggests that the LLIV strain constitutively uses the GDH pathway in addition to the GS-GOGAT pathway to maintain intracellular nitrogen levels, consistent with adaptation of the LLIV clade to an elevated-nutrient, energy-poor environment in the deep euphotic zone. Rangel et al. (51) showed that the GDH pathway likely operates in reverse in MIT9313 to transfer ammonium from glutamate, providing a route for utilizing amino acids as a nitrogen source, consistent with findings of elevated mixotrophy in LL-adapted ecotypes (56, 57). Intriguingly, MIT0801 (LLI) also accumulated high intracellular concentrations of glutamic acid, a hint that this strain could be using a combination of nitrogen assimilation strategies (Fig. 4).

Another sign that MIT9313 is accustomed to a relatively high-nitrogen environment is its unique ability to synthesize nitrogen-rich organic compatible solutes to offset the inorganic saline ocean. Specifically, we detected very high concentrations of glycine betaine, a common microbial N-containing osmolyte, in MIT9313, consistent with genomic evidence that this strain is uniquely capable of glycine betaine synthesis among our chosen strains and with previous work showing glycine betaine accumulation in MIT9313 (58). In the current study, intracellular glycine betaine concentrations reached 3 ± 1.3 fg cell^−1^ or 4%–6% of its total biomass (Table S3), supporting its role as an osmolyte in this strain (59). In contrast to MIT9313, *Prochlorococcus* ecotypes living higher in the water column use nitrogen-free osmolytes such as sucrose and glucosyl-glycerate over glycine betaine (59). The methods here do not capture dissolved glycine betaine efficiently, so this compound is not quantified in the extracellular metabolite fraction. Nevertheless, if glycine betaine production by MIT9313 leads to its excretion, it could promote interactions with neighboring heterotrophs (60), many of which have high-affinity transporters for glycine betaine (61) including the most ubiquitous marine heterotrophic bacteria, SAR11 (62). Indeed, studies of co-cultures of *Prochlorococcus* and SAR11 showed that MIT9313, but not strains from other clades, could fulfill the glycine requirement for SAR11 growth (63), consistent with excretion of glycine betaine.

### Metabolite differences within the branched-chain amino acid (BCAA) pathways

**Fig 5:**
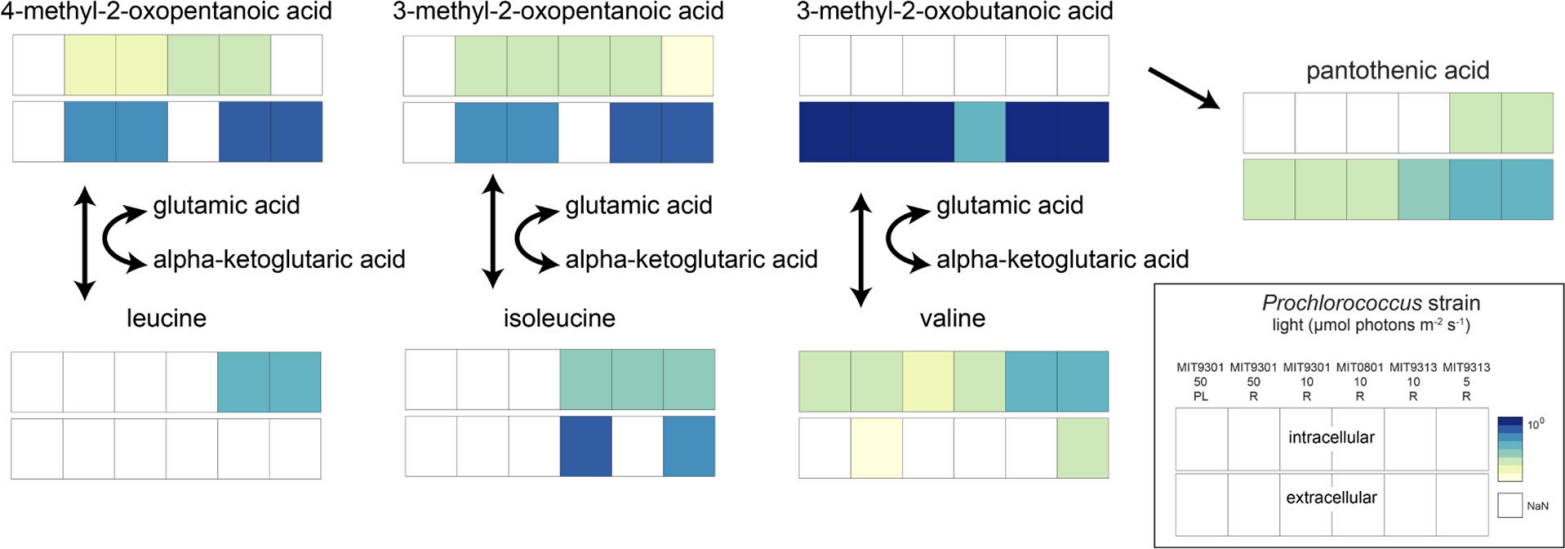
Branched-chain amino acid pathway figure with intermediates and BCAAs. Squares are log values of mean from Fig. 1 (top, intracellular; bottom, extracellular). A bar graph representation of these data is provided in Fig. S2.

The BCAAs, valine, leucine and isoleucine, and their precursors, were consistently among the most abundant metabolites measured (Fig. 5; Fig. S3). When grown under nutrient-replete conditions, MIT9301 and MIT9313 excreted high levels of 3-methyl-2-oxobutanoic acid (precursor to valine), 3-methyl-2-oxopentanoic acid (precursor to isoleucine), and 4-methyl-2-oxopentanoic acid (precursor to leucine), but when grown under P-limitation, MIT9301 excreted only 3-methyl-2-oxobutanoic acid. MIT0801, grown under nutrient-replete conditions, also excreted only 3-methyl-2-oxobutanoic acid among the three branched-chain precursors. These observations suggest that *Prochlorococcus* modulates excretion of 3-methyl-2-oxopentanoic acid and 4-methyl-2-oxopentanoic acid under some growth conditions but maintains excretion of 3-methyl-2-oxobutanoic acid. However, as a point of difference in these pathways, in MIT0801, isoleucine was among the most abundant extracellular metabolites, but this compound was undetectable in the other *Prochlorococcus* strains. In general, BCAAs were either not detected or had much lower concentrations than their precursors in MIT9301 and MIT9313, except for isoleucine in MIT9313 grown at low light, where it reached similar levels as its precursor.

Perhaps, a more fundamental question regarding the BCAA precursors is why *Prochlorococcus* excretes these compounds in the first place. BCAAs play important roles in protein structure due to their hydrophobic and sterically hindered functionalities, and their synthesis pathways are classic examples of allosteric regulation due to the many feedbacks within these pathways (e.g., references 64, 65). In all bacteria, BCAA synthesis pathways culminate in the transfer of an amine group from glutamic acid to a BCAA precursor, catalyzed by a shared transaminase. This enzyme is reversible and is the first step in BCAA catabolism. *Prochlorococcus* genomes do not contain the full catabolic pathway, but they retain the transaminase step for BCAA amination and de-amination. Consequently, *Prochlorococcus* cannot regulate intracellular BCAA concentrations by catabolizing BCAAs, a common strategy in other microbes (65). Our data cannot distinguish between excretion of these precursors prior to BCAA production or after the first step of BCAA catabolism. We considered the possibility that excretion of BCAA precursors reflects a form of “directed overflow” metabolism (66), a homeostatic control mechanism in which cells maintain a robust supply of pathway intermediates to ensure efficient synthesis of end-products, and excrete any excess they cannot reuse or recycle. However, in that scenario, we might also expect to observe high intracellular levels of the BCAA precursors, but they accumulated at lower levels, if at all, than the BCAAs in all three *Prochlorococcus* strains. The contrast was particularly stark for 3-methyl-2-oxobutanoic acid, which was never detected intracellularly, but its product valine was present in all strains. This may indicate that BCAA intracellular concentrations are regulated to some extent by catabolism to their precursors (to retain intracellular nitrogen), followed by excretion.

Excretion of the nitrogen-free BCAA precursors could, therefore, reflect large-scale forces shaping *Prochlorococcus* metabolism over the course of its evolution in the nutrient-poor open ocean. Reconstructions in the core of carbohydrate metabolism suggest that *Prochlorococcus* has added several pathways for excreting organic carbon as a necessary by-product of increasing the cellular ATP:ADP ratio to compensate for the increased free energy cost of transporting nutrients from low background concentrations (15). In this context, we note that 3-methyl-2-oxobutanoic acid is derived from two molecules of pyruvate, which was identified as one of the central outlets of organic carbon excretion in *Prochlorococcus* (15).

While our observations suggest links between cellular state and the dynamics of BCAAs, some observed differences across strains hint that other factors and differences between ecotypes are at play. For example, MIT9313 (LLIV) releases the highest levels of 3-methyl-2-oxobutanoic acid and accumulates the highest intracellular concentrations of valine. The justification for high concentrations of valine (intracellular) and its precursor (extracellular) in the LLIV ecotype, relative to the other strains, is unknown. BCAA precursors could be valuable metabolites to nearby microbes in the field because they require just the transfer of an amino group to generate BCAAs or because their catabolism can shuttle carbon into core energy-producing pathways. In co-culture experiments with *Prochlorococcus* NATL2A (LLI) and the heterotroph *Alteromonas macleodii*, transcripts for valine catabolism were upregulated in *A. macleodii* relative to a control (67), consistent with cross-feeding of valine and/or its precursor. Although we detected valine in the extracellular phase of two of our strains, this result is treated cautiously due to analytical challenges with the measurement of dissolved valine. Moreover, marine bacteria have not yet been shown to be auxotrophic for BCAAs or their precursors. Nevertheless, our data suggest that these molecules are released at high levels by some *Prochlorococcus* strains and may serve as sources of reduced carbon and/or circumvent the full biosynthetic pathways for BCAAs in nearby heterotrophic bacteria.

**Fig 6:**
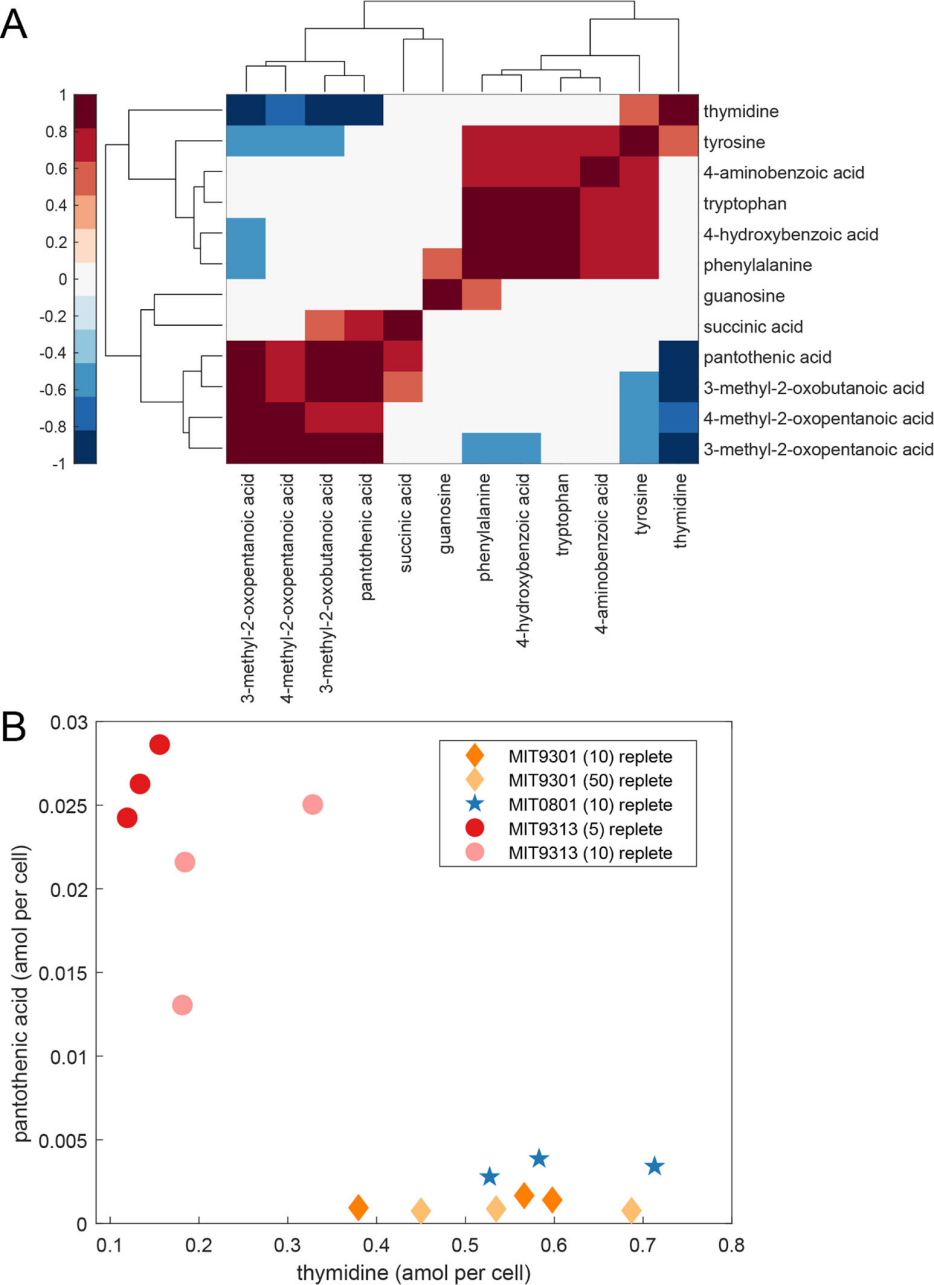
(**A**) Clustergram of correlations for extracellular (dissolved) metabolites occurring in at least three of five treatments. The complete set of correlations for dissolved metabolites is provided in Fig. S4. Statistically significant positive (red) and negative (blue) correlations are Pearson correlations with *P*-values adjusted using a false discovery rate of 5%. (**B**) Dissolved pantothenic acid plotted against dissolved thymidine, which are significantly negatively correlated (orange, MIT9301; blue, MIT0801; red, MIT9313).

When we considered the extracellular dynamics of the BCAA precursors, we noticed that the extracellular concentrations of BCAA precursors were positively correlated with one another and with pantothenic acid (vitamin B_5_; *R* = 0.9, *P*-value <0.001 for pantothenic acid and 3-methyl-2-oxobutanoic acid; Fig. 6), while also negatively correlated with thymidine (*R* = −0.85, *P*-value <0.001 for pantothenic acid and thymidine; Fig. 6). MIT9313, the LLIV strain, releases higher concentrations of pantothenic acid and BCAA precursors, relative to thymidine. The other two strains have the opposite dynamic, with relatively high thymidine release and lower excretion of pantothenic acid and BCAA precursors. The negative correlation of the three BCAA precursors and pantothenic acid with dissolved thymidine concentrations cannot be explained with any known mechanism. These correlations likely combine differences based on phylogeny with differences in light growth conditions, so it is particularly challenging to identify one explanation. Links between BCAA and thymidine metabolism have been observed in the context of plant herbicide research (68, 69), but these connections require thymidine kinase, an enzyme that is absent in all *Prochlorococcus* genomes. Historically, thymidine was used to assay heterotrophic productivity in the ocean (70) because many microbes can use this compound to make nucleic acids. High levels of thymidine release by *Prochlorococcus* indicate that its cross-feeding may be important in the oligotrophic oceans, warranting further study.

Pantothenic acid provides the scaffold for coenzyme A (CoA), a central metabolite in carbon oxidation cycles, and is one of the most ancient B vitamins due to its ubiquitous role in carbon cycling (71). Furthermore, pantothenic acid is a downstream product of 3-methyl-2-oxobutanoic acid, raising the possibility of a link between the exudation of these compounds, although the underlying mechanism remains unclear. We observed intracellular pantothenic acid only in MIT9313, and as it would likely be quickly converted to CoA, the factors behind its release are not known. However, some ubiquitous marine heterotrophs are auxotrophic for pantothenic acid, including SAR11 and SAR86 (72), suggesting that this molecule may serve an important role in establishing relationships between *Prochlorococcus* and globally abundant sympatric heterotrophic microbes in the surface ocean. Although pantothenic acid has been observed in other culture exudates (73), little is known of its concentrations or dynamics in marine systems (74, 75). The connections observed here among BCAA precursors, pantothenic acid, and thymidine hint at potential currencies that could mediate interactions between *Prochlorococcus* and its heterotrophic neighbors.

### Further implications

Stepping back and examining extracellular metabolites in their entirety (Fig. S4), we identified additional metabolites that may influence microbial interactions in the ocean. Here, we constrained our interpretation of dissolved metabolites to those whose extraction efficiencies were >1% (76) and were present in the cultures of at least two strains. In addition to those noted above as present in all strains, five metabolites were detected in the dissolved phase along strain-specific delineations. 5′-Methylthioadenosine (MTA), a by-product of polyamine metabolism and the precursor to methionine salvage, was released by MIT9313 under nutrient-replete conditions—albeit at low concentrations—and by MIT9301 under P-limited growth conditions. Release of MTA by MIT9313 is somewhat surprising, as MTA is recycled via the methionine salvage pathway, which is complete only in MIT9313. We hypothesize that *Prochlorococcus* outside of the LLIV clade (including MIT0801 and MIT9301) might instead release 5-methyl-thioribose-1-phosphate (MTRP), a downstream product of MTA that forms a dead end in the network. In this scenario, MTA is released by MIT9301 due to a bottleneck in the availability of phosphate, preventing its conversion to MTRP. MTRP could make for an interesting target in future studies, although it is not currently commercially available and is difficult to synthesize.

We observed release of metabolites whose syntheses are not annotated in any strain’s genome, specifically, 2,3-dihydroxypropane sulfonate (DHPS), xanthine, and kynurenine. All three molecules may be spontaneous reaction products from oxidation or degradation reactions intended for other metabolites. Xanthine and DHPS may be products of nucleic acid degradation (77) and sulfolipid synthesis (78 – 80), respectively. As noted above, kynurenine is predicted to result from ROS-mediated oxidation of tryptophan (41). Each of these metabolites could be a high-value compound for neighboring heterotrophic bacteria, and further work is needed to ascertain whether specific relationships could develop based on these molecular exchanges.

More generally, a notable result in this study is the large variability among metabolite profiles from the three *Prochlorococcus* strains grown under replete conditions. Early metabolomics studies often used one representative strain of model microorganisms for assessing characteristic metabolomes, due to the challenges in collecting and analyzing these data. Indeed, these studies showed that metabolite profiles can reflect differences between organisms with significant genomic variability (e.g., reference 25). However, our data and previous work (26) suggest that individual metabolite profiles can also be quite different among microbes with more similar genomes. Some metabolite concentrations differed among *Prochlorococcus* strains by a factor of 10× or more in both intracellular and extracellular phases, when normalized by cell number (Fig. 1), which suggests differences in regulation, overflow metabolism (66), or other processes that govern how these strains interact with their environment. Some of these differences are larger than those observed in studies within one strain when changing growth conditions (including nutrient limitation [55, 81]), underscoring the importance of assessing cross-strain differences in addition to within-strain responses to anticipated environmental stressors.

In addition to cross-strain differences, metabolite profiles were generally uncoupled between intracellular and extracellular phases. Direct comparisons between intra- and extracellular phases should be undertaken carefully because the intracellular profiles reflect near-time phenotypes, while the extracellular profiles are the result of accumulation over time in the growth medium. However, if we focus this comparison on metabolites that are equally detectable between the phases (Table S2), we observe cases in which metabolites increase (or decrease) in tandem between the phases (e.g., phenylalanine and pantothenic acid), consistent with internal accumulation driving excretion. However, we also see striking differences in the two phases for many metabolites. For example, glutathione and tyrosine are retained within *Prochlorococcus* cells but are detected only in some strains in the extracellular phase. In contrast, thymidine and 3-methyl-2-oxobutanoic acid are not detected intracellularly but are present at high attomole per cell concentrations in the dissolved phase. Thus, *Prochlorococcus* release these metabolites to the external environment without retaining detectable concentrations inside cells. These metabolites could be important currencies in consortia containing *Prochlorococcus*, as they are immediate precursors to nucleic acids, BCAAs, and vitamins. From work with single marine microbial strains, we know that external metabolite profiles are affected by nutrient status (82) and likely by the presence or absence of sympatric heterotrophs (83).

This study represents the first measurements of some of the metabolites we report and underscores the need for additional metabolomics experiments that describe metabolic differences among closely related microbial strains. We observed distinct intracellular and extracellular metabolic profiles arising from the three ecotypes we evaluated, implying that *Prochlorococcus* at different depths in the euphotic zone will have distinct impacts on the metabolism and dissolved organic matter (DOM) dynamics in these regions. Some of these differences occurred within central metabolic pathways, limiting the ability of genomics information to predict the intracellular metabolic profiles of these organisms. As noted above, we observed de-coupling between the intracellular and extracellular metabolites in many cases, suggesting that molecules excreted into the environment cannot be fully anticipated from intracellular measurements. More work is needed on many of these metabolites, as there is insufficient background knowledge on these compounds’ biochemistries to anticipate the roles they may play in connecting microbes within the surface ocean microbiome. In parallel to biochemical studies, more dissolved metabolite analyses will be needed to establish the dominant currencies of the marine microbial loop. Some of the molecules observed and described here are excellent targets for field, laboratory, and modeling efforts.

## MATERIALS AND METHODS

### Strain information

The strains in this study included MIT9301 (HLII) (84), MIT0801 (LLI) (33), and MIT9313 (LLIV) (8). Genomes for each of these strains are publicly available, and the KEGG contains information on their known biochemical pathways: MIT9301, “pmg” in KEGG; MIT0801, “prm”; and MIT9313, “pmt.”

### Culture conditions

We cultured all three strains individually in 0.2-µm filter-sterilized Sargasso Sea water amended with sterile Pro99 nutrients (85) at 24°C under constant illumination (Sylvania Cool white bulbs screened to provide required light levels). Constant illumination results in cells that are asynchronous, and thus, measurements from each time point reflect an average over the full diel cycle. We chose light levels to balance maximizing growth rates *in vitro* (5, 8) and conditions relevant to different ecotypes in the wild (10) and to facilitate light-independent comparisons among strains, i.e., when possible growing all three strains at the same light intensity (Table 1). For example, all three strains were grown at 10 µmol photons m^−2^ s^−1^, which lies between the optimal level to achieve maximum *in vitro* growth rates for members of the LLI ecotype (MIT0801) and conditions in the mid-euphotic zone where populations of the LLI ecotype typically thrive. Since the HLII and LLIV ecotypes generally inhabit the upper and lower euphotic zone, respectively (10), we also acclimated MIT9301 to a higher irradiance (50 µmol photons m^−2^ s^−1^) and MIT9313 to a lower irradiance (5 µmol photons m^−2^ s^−1^).

To assess the impact of nutrient limitation, we grew MIT9301 at the higher irradiance (50 µmol photons m^−2^ s^−1^) under semi-continuous phosphate limitation through daily transfers (at a dilution rate of 0.3 day^−1^) in media with 20-fold lower phosphate concentrations (i.e., 2.5 µM phosphate vs 50 µM phosphate) than the nutrient-balanced Pro99 media used for the other cultures. We maintained each strain at its target irradiance levels (±5%) for >50 generations in triplicate axenic batch cultures in acid-washed autoclaved borosilicate glass tubes before sampling. We confirmed purity by flow cytometry and a suite of purity test broths (ProAC, ProMM, and MPTB) as previously described (5, 22, 86 – 89). We monitored growth via bulk fluorescence measurements and enumerated cells using a Guava Technologies easyCyte 12HT flow cytometer (EMD Millipore) measuring red fluorescence (695/50) and forward scatter from blue (488 nm) excitation. We diluted samples in sterilized Sargasso Sea water prior to enumeration to ensure <500 cells µL^−1^ and avoid coincidence counting.

### Metabolite sampling and extractions

We sampled triplicate batch cultures (35 mL) in mid-exponential growth for both intracellular and extracellular metabolite profiles for all replete treatments. For the P-limited MIT9301 cultures, cell densities were significantly lower due to the phosphate limitation, requiring us to sample over several days—as dictated by the dilution rate—to obtain an approximately similar total number of cells as from the replete cultures. Specifically, we sampled 9 mL (i.e., the volume of the culture replaced with fresh media each day) from the semi-continuous cultures for 6 consecutive days before destructively sampling the full remaining volume. All daily samples from each replicate culture were combined after completion of the experiment. Due to this “averaging” effect of combining the daily samples and the more complex sampling scheme, we opted to obtain duplicates rather than triplicates for the P-limited cultures. All glassware used in metabolite extractions was acid washed, rinsed with ultrapure water (EMD Millipore), and combusted (450°C for 8 h) prior to use. We filtered each replicate through a 0.1-µm Omnipore filter (Millipore) in a glass filtration tower under a gentle vacuum, and exact volumes were recorded with sterile serological pipettes. We used the filters for intracellular metabolites; they were folded (sample side in), placed in cryovials, and immediately stored at −80°C. We used the filtrates for extracellular metabolites; they were transferred to acid-washed, autoclaved polycarbonate bottles and acidified with 1 µL of 12 M HCl per milliliter of sample (final pH ≈ 3) before storage at −20°C.

We extracted intracellular metabolites using a method described by Rabinowitz and Kimball (90) and modified as described in Kido Soule et al. (91). Briefly, we extracted each filter three times with ice-cold extraction solvent (40:40:20 acetonitrile:methanol:water with 0.1 M formic acid). We neutralized the combined extracts with 6 M ammonium hydroxide and dried them in a vacufuge until near dryness.

We extracted extracellular metabolites from the seawater matrix using Bond Elut PPL cartridges (1 g/6 mL sized cartridges; Agilent) following the protocol of Dittmar et al. (92) as modified by Longnecker (93). We eluted dissolved metabolites from the cartridges with 100% methanol and stored them at −20°C until analysis. Immediately prior to analysis, we dried (to near dryness) the extracts with a vacufuge.

### Targeted mass spectrometry

We re-constituted all extracts (intracellular and extracellular) in 95:5 (vol/vol) water:acetonitrile with isotopically labeled injection standards (50 pg/µL each; d_2_-biotin, d_6_-succinic acid, d_4_-cholic acid, d_7_indole-3-acetic acid, and ^13^C_1_-phenylalanine). To quantify targeted metabolites, we used ultra-performance liquid chromatography (Accela Open Autosampler and Accela 1250 Pump, Thermo Scientific) coupled to a heated electrospray ionization source and a triple quadrupole mass spectrometer (TSQ Vantage, Thermo Scientific) operated under selected reaction monitoring (SRM) mode. We performed chromatographic separation with a Waters Acquity HSS T3 column (2.1 × 100 mm, 1.8 µm) equipped with a Vanguard pre-column and maintained at 40°C. We eluted the metabolites from the column with (A) 0.1% formic acid in water and (B) 0.1% formic acid in acetonitrile at a flow rate of 0.5 mL min^−1^, according to the gradient: 0 min, 1% B; 1 min, 1% B; 3 min, 15% B; 6 min, 50% B; 9 min, 95% B; 10 min, 95% B; 10.2 min, 1% B; 12 min, 1% B (total run time, 12 min). For positive and negative modes, we performed separate autosampler injections of 5 µL each.

We separated extracts into intra- and extracellular batches and generated a pooled sample using 25 µL of each sample. Within a batch, we analyzed the samples in random order. We operated the mass spectrometer in SRM mode with optimal SRM parameters (s-lens, collision energy) for each target compound determined previously with an authentic standard. The complete list of metabolites is provided in Table S2. We monitored two SRM transitions per compound for quantification and confirmation and generated 10-point external calibration curves based on peak area for each compound. We converted raw data files from proprietary Thermo (.RAW) format to mzML using the msConvert tool (94) prior to processing with MAVEN (95). We required metabolites to be detected in two of three biological replicates (or in both replicates of the P-limited MIT9301 cultures) to be considered for further analysis. Extracellular metabolite concentrations were corrected for extraction efficiency (76). We report all detected metabolites but only concentrations for those with extraction efficiencies >1%. Extracellular (dissolved) and intracellular (particulate) metabolite abundances are presented as cell-specific concentrations, calculated as the moles of each metabolite divided by *Prochlorococcus* cell number.

### Statistics, data, and code availability

We confirmed that the target metabolites in this study could be synthesized by at least one strain using the KEGG module within Biopython (96). Because KEGG is not organized to consider strain-specific differences in metabolites, we queried KEGG to find the genes unique to each strain, then to determine the list of reactions associated with a given gene, and finally, to identify chemical compounds (metabolites) associated with each pathway.

For MIT9301 and MIT9313, *Prochlorococcus* was grown at two light levels, and we tested for differences due to light with a *t*-test where *P*-values were adjusted for multiple comparisons allowing a false discovery rate of 5% (97). Correlations between metabolites were calculated as Pearson correlations, and *P*-values were adjusted using a false discovery rate of 5%. A two-way analysis of variance using log_10_-transformed data followed by post-hoc tests using Fisher’s least significant difference was used to consider differences in the ratio of metabolites across ecotype and light level. Clustergrams were calculated in MATLAB 2019b using a Euclidean distance metric and average linkage. Select figures were made using the notBoxPlot function from the MATLAB File Exchange; these figures show discrete datapoints, the mean value, and the 95% confidence interval for the mean.

## Data Availability

Targeted metabolomics data for this project are available from MetaboLights (https://www.ebi.ac.uk/metabolights/MTBLS567). The code to query genetic data based on metabolomics information is available at GitHub (https://github.com/KujawinskiLaboratory/Pro_mtabs/blob/master/find_metabolites_by_strain.ipynb).

## SUPPLEMENTAL MATERIAL

Tables S1 to S3; Figures S1 to S4 msystems.01261-22-s0001.docx Extra tables on growth rates, metabolites and carbon factors; extra Figures showing P-limited growth, metabolite concentrations, and clustergram of extracellular metabolites.

ASM does not own the copyrights to Supplemental Material that may be linked to, or accessed through, an article. The authors have granted ASM a non-exclusive, world-wide license to publish the Supplemental Material files. Please contact the corresponding author directly for reuse.
