# Erratum for Kujawinski et al., “Metabolite diversity among representatives of divergent *Prochlorococcus* ecotypes”

**DOI:** 10.1128/msystems.00120-24

**Published:** 2024-02-27

**Authors:** Elizabeth B. Kujawinski, Rogier Braakman, Krista Longnecker, Jamie W. Becker, Sallie W. Chisholm, Keven Dooley, Melissa C. Kido Soule, Gretchen J. Swarr, Kathryn Halloran

## ERRATUM

Volume 8, no. 5, e01261-22, 2023, https://doi.org/10.1128/msystems.01261-22. Page 17, Acknowledgments: The following should be added. “This is C-CoMP publication no. 032.”

Page 18, Author Contributions: The following should be added. “Elizabeth Kujawinski, Conceptualization, Formal analysis, Funding acquisition, Investigation, Project administration, Resources, Visualization, Writing – original draft, Writing – review and editing.”

