# Supplementary material for: Metabolite diversity among representatives of divergent Prochlorococcus ecotypes
Source: mSystems. 2023 Oct 10;8(5):e01261-22. doi: 10.1128/msystems.01261-22 (PMC10654061; doi:10.1128/msystems.01261-22)
Supplement: Tables S1 to S3; Figures S1 to S4 — Extra tables on growth rates, metabolites and carbon factors; extra Figures showing P-limited growth, metabolite concentrations, and clustergram of extracellular metabolites. [file msystems.01261-22-s0001.docx]

Metabolite diversity among representatives of divergent *Prochlorococcus* ecotypes

# Supplemental information

## Table S1.

## Table of growth rates at each light intensity for each strain. Growth rates are averages of the five transfers before the onset of the experiment.

| **Strain** | **Light intensity**  **(µmol photons m^-2^ s^-1^)** | **Phosphate levels** | **Average growth rate (day^-1^)** |
| --- | --- | --- | --- |
| MIT9301 | 50 | low phosphate | 0.29 |
| MIT9301 | 10 | phosphate-replete | 0.39 |
| MIT9301 | 50 | phosphate-replete | 0.67 |
| MIT0801 | 10 | phosphate-replete | 0.40 |
| MIT9313 | 5 | phosphate-replete | 0.29 |
| MIT9313 | 10 | phosphate-replete | 0.43 |

## Table S2.

Complete set of metabolites within the targeted metabolomics method used in the current project and the extraction efficiency information from Johnson et al. (1) which have been updated with unpublished data. Metabolites marked with ‘yes’ in intracellular and/extracellular columns were found in at least one *Prochlorococcus* strain under any light condition. We report all detected metabolites, but only concentrations for those with extraction efficiencies above 1%. Intracellular concentrations of oxidized glutathione are not available due to interference by an unknown compound. The concentration data for each metabolite is available at MetaboLights (http://www.ebi.ac.uk/metabolights/) as study accession number MTBLS567.

| **metabolite** | **extraction efficiency (%)** | **intracellular** | **extracellular** |
| --- | --- | --- | --- |
| 2,3-dihydroxybenzoic acid | 100.9 |  |  |
| 2,3-dihydroxypropane-1-sulfonate | 0.6 |  | yes |
| 3-mercaptopropionic acid | 88.6 |  |  |
| 3-methyl-2-oxobutanoic acid | 10.1 |  | yes |
| 3-methyl-2-oxopentanoic acid | 50.1 | yes | yes |
| 4-aminobenzoic acid | 18.5 | yes | yes |
| 4-hydroxybenzoic acid | 88 |  | yes |
| 4-methyl-2-oxopentanoic acid | 43.4 | yes | yes |
| 5'-methylthioadenosine | 80.9 | yes | yes |
| 4-amino-5-aminomethyl-2-methylpyrimidine | 0 |  |  |
| D-glucosamine 6-phosphate | 0 |  |  |
| dimethylsulfoniopropionate | 0 |  |  |
| γ-aminobutyric acid | 0 |  |  |
| 4-Amino-2-methyl-5-pyrimidinemethanol | 0 |  |  |
| nicotinamide adenine dinucleotide | 20.9 | yes |  |
| putrescine | 0 |  | yes |
| acetyltaurine | 0 |  |  |
| adenine | 0 |  |  |
| adenosine | 6.5 |  |  |
| adenosine 5'-monophosphate | 0.2 | yes |  |
| alpha-ketoglutaric acid | 0 | yes |  |
| arginine | 0 | yes |  |
| aspartic acid | 0 | yes |  |
| glycine betaine | 0 | yes |  |
| biotin | 52.8 |  |  |
| caffeine | 23.5 |  |  |
| chitobiose | 0.3 | yes |  |
| chitotriose | 5.6 |  |  |
| choline | 0 |  |  |
| ciliatine | 0 |  |  |
| citrate | 0.9 |  |  |
| citrulline | 0 | yes |  |
| cyanocobalamin | 79.1 |  |  |
| cysteine | 0 |  |  |
| cytosine | 0 |  |  |
| desthiobiotin | 6.5 |  |  |
| dihydroxyacetone phosphate | 0 | yes |  |
| ectoine | 0 |  |  |
| folic acid | 41.2 |  |  |
| fosfomycin | 0 |  |  |
| fumaric acid | 0 |  |  |
| glucose 6-phosphate | 0 | yes |  |
| glutamic acid | 0 | yes |  |
| glutamine | 0 | yes |  |
| glyphosate | 15.1 |  |  |
| glutathione | 1.1 | yes | yes |
| glutathione oxidized | 1.5 | not available | yes |
| guanine | 0 |  |  |
| guanosine | 7.7 | yes | yes |
| hemin | 0 | yes |  |
| indole 3-acetic acid | 16.8 |  |  |
| inosine | 8.1 |  |  |
| inosine 5'-monophosphate | 0 | yes |  |
| isoleucine | 1.41 | yes | yes |
| kynurenine | 41.7 |  | yes |
| leucine | 3.3 | yes |  |
| malic acid | 0.7 |  |  |
| methionine | 0 | yes |  |
| muramic acid | 0 |  |  |
| n-acetyl glucosamine | 0 |  |  |
| n-acetyl glutamic acid | 1.1 | yes |  |
| n-acetyl muramic acid | 2.8 |  |  |
| ornithine | 0 |  |  |
| orotic acid | 0 |  |  |
| pantothenic acid | 51.9 | yes | yes |
| phenylalanine | 39.7 | yes | yes |
| phosphoenolpyruvate | 0 |  |  |
| phycocyanobilin | 0 |  |  |
| proline | 0 | yes |  |
| pyridoxine | 6.8 |  |  |
| riboflavin | 87.6 |  |  |
| S-(5'-adenosyl)-L-homocysteine | 43.3 | yes |  |
| S-adenosyl-L-methionine | 0 | yes |  |
| alanine (isom. sarcosine) | 0 |  |  |
| serine | 0 |  |  |
| sn-glycerol 3-phosphate | 0 |  |  |
| taurocholic acid | 92.8 |  |  |
| spermidine | 0 | yes | yes |
| succinic acid | 0 |  | yes |
| syringic acid | 32.9 |  |  |
| taurine | 0 |  |  |
| thiamine | 2 |  |  |
| thiamine monophosphate | 0 |  |  |
| threonine / homoserine | 0 |  |  |
| thymidine | 52.6 |  | yes |
| tryptamine | 21.3 |  |  |
| tryptophan | 46.7 | yes | yes |
| tyrosine | 2.1 | yes | yes |
| uracil | 0 | yes |  |
| uridine 5'-monophosphate | 0 | yes |  |
| valine | 0 | yes | yes |
| xanthine | 0.3 |  | yes |
| xanthosine | 10.4 |  | yes |

## Table S3.

Mean (± one standard deviation) cell-specific concentrations of glycine betaine (fg cell^‑1^) in cultures of three strains of *Prochlorococcus* grown at a range of light intensities. *Biomass of cells from each strain are from Cermak et al. (2) in which the authors used a microfluidic mass sensor. We assumed 50% of the cell was carbon (3). These values were used to calculate the percent of each strain’s intracellular carbon content that can be attributed to the carbon in glycine betaine. ^†^Measured value for NATL2A, a related LLI strain of *Prochlorococcus*.

| **Strain** | **Clade** | **Growth light intensity (µmol photons m^-2^s^-1^)** | **Median cellular biomass (fg)*** | **Glycine betaine**  **(fg cell^-1^)** | **% of biomass** |
| --- | --- | --- | --- | --- | --- |
| MIT9301 | HLII | 10 | 60 ± 3 | 4.5 x 10^-4^ (± 8.0 x 10^-4^) | 0.002% |
| MIT9301 | HLII | 50 | 60 ± 3 | 0 |  |
| MIT0801 | LLI | 10 | 91 ± 5^†^ | 0 |  |
| MIT9313 | LLIV | 5 | 158 ± 6 | 3.0 (± 1.3) | 4% |
| MIT9313 | LLIV | 10 | 158 ± 6 | 4.7 (± 2.4) | 6% |

***Supplemental figures:***


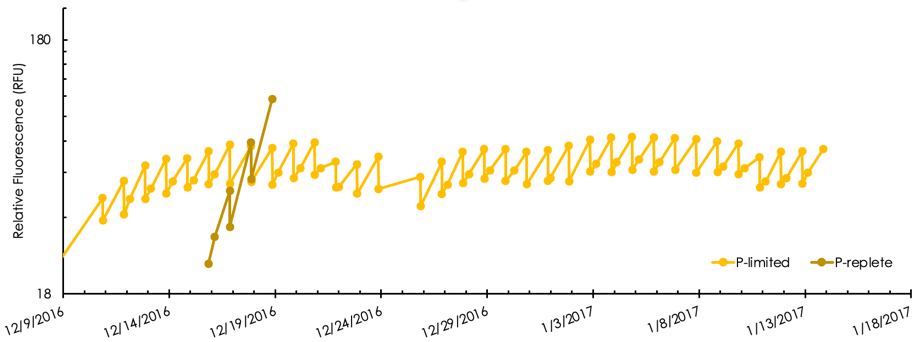


**Figure S1:** The yellow curve shows a culture of *Prochlorococcus* MIT9301 maintained in a semi-continuous state of P-limitation through daily dilutions with media containing 20-fold less phosphorus than the replete media. The brown curve shows a culture that from that point forward was diluted (at the same dilution rate) in media replete with phosphorus.


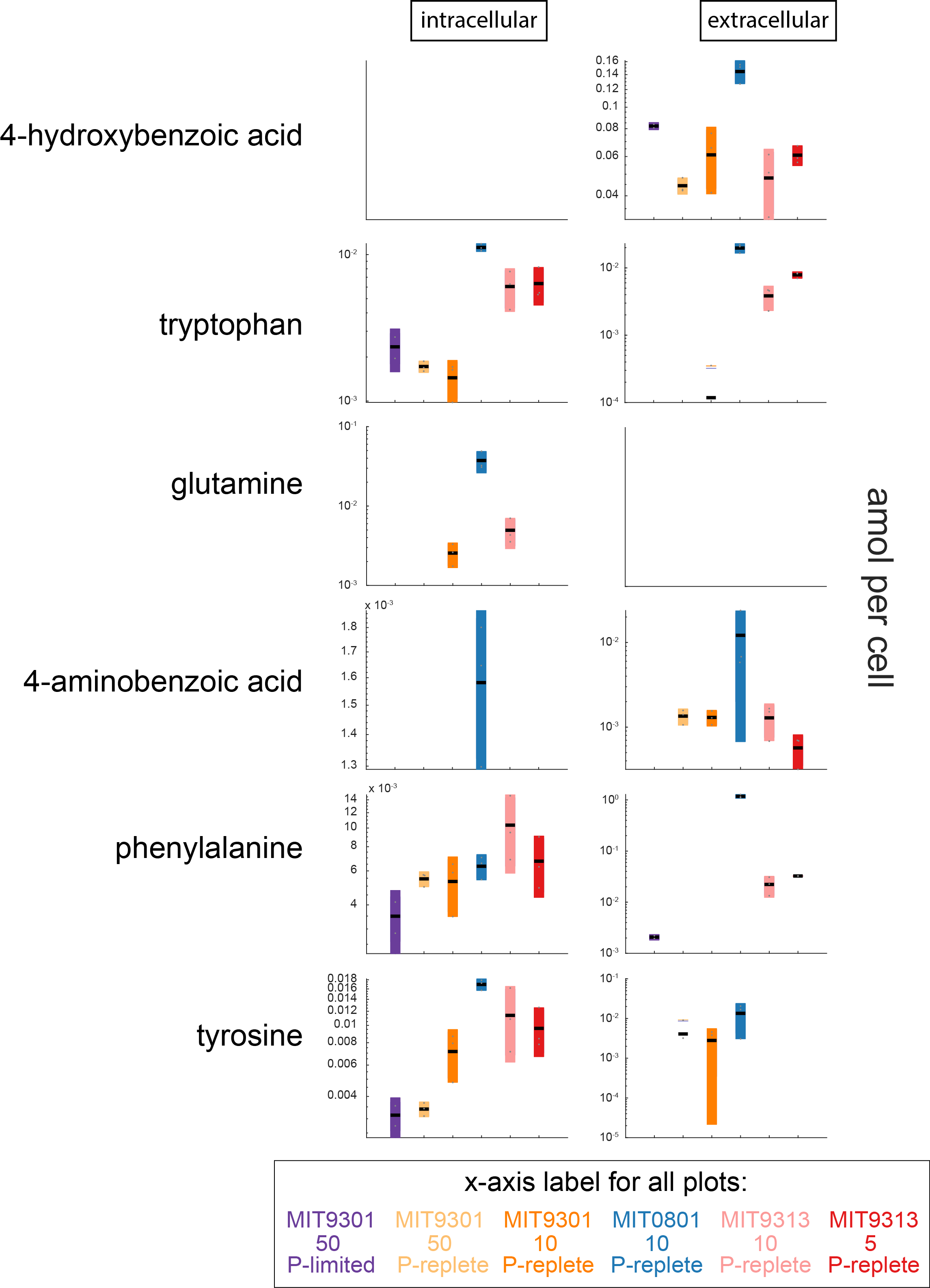
**Figure S2.** Intracellular metabolites from Figure 3, plotted on a log scale as discrete amol per cell values for each metabolite.


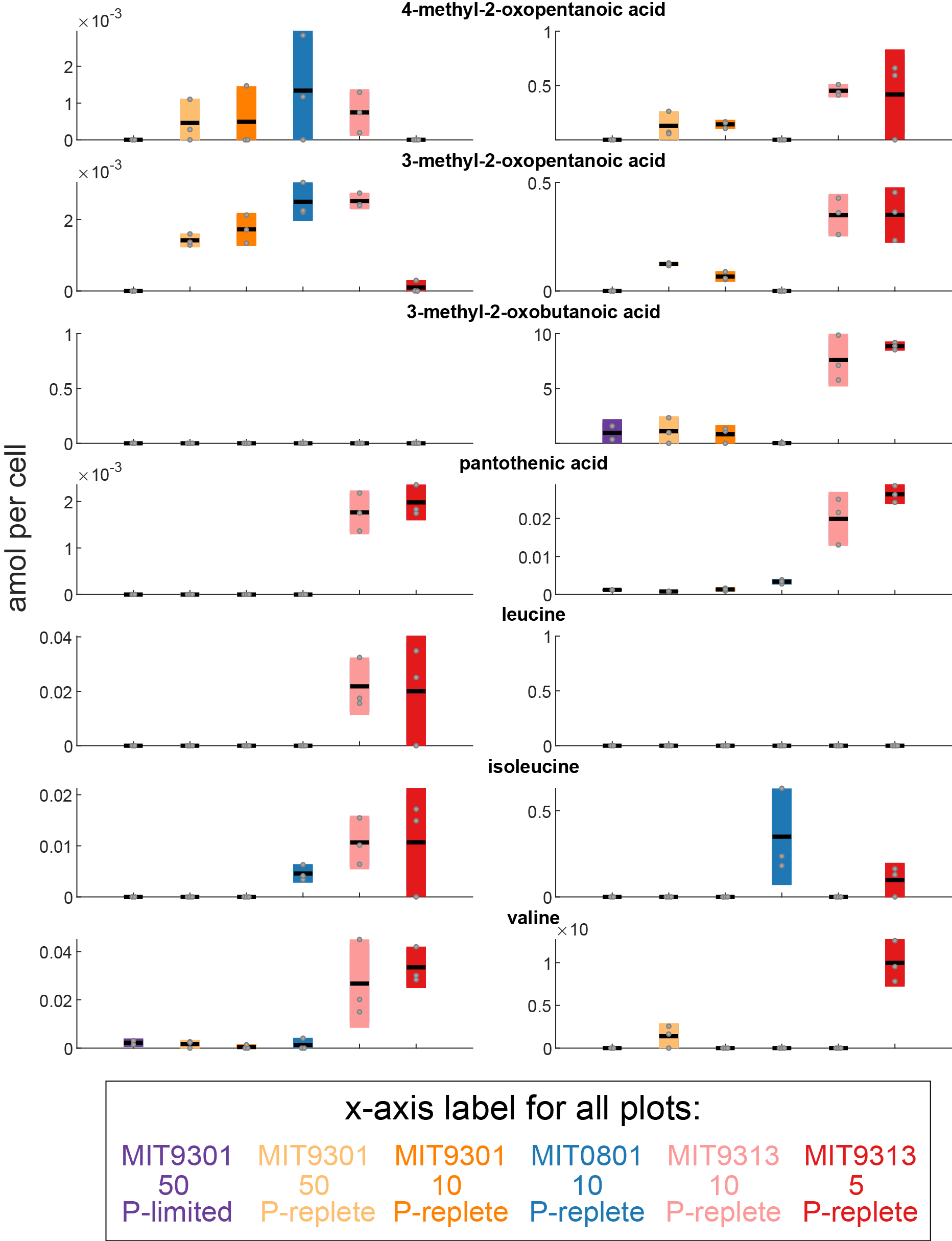
**Figure S3.** Intracellular metabolites from Figure 5, plotted as discrete amol per cell values for each metabolite.

**
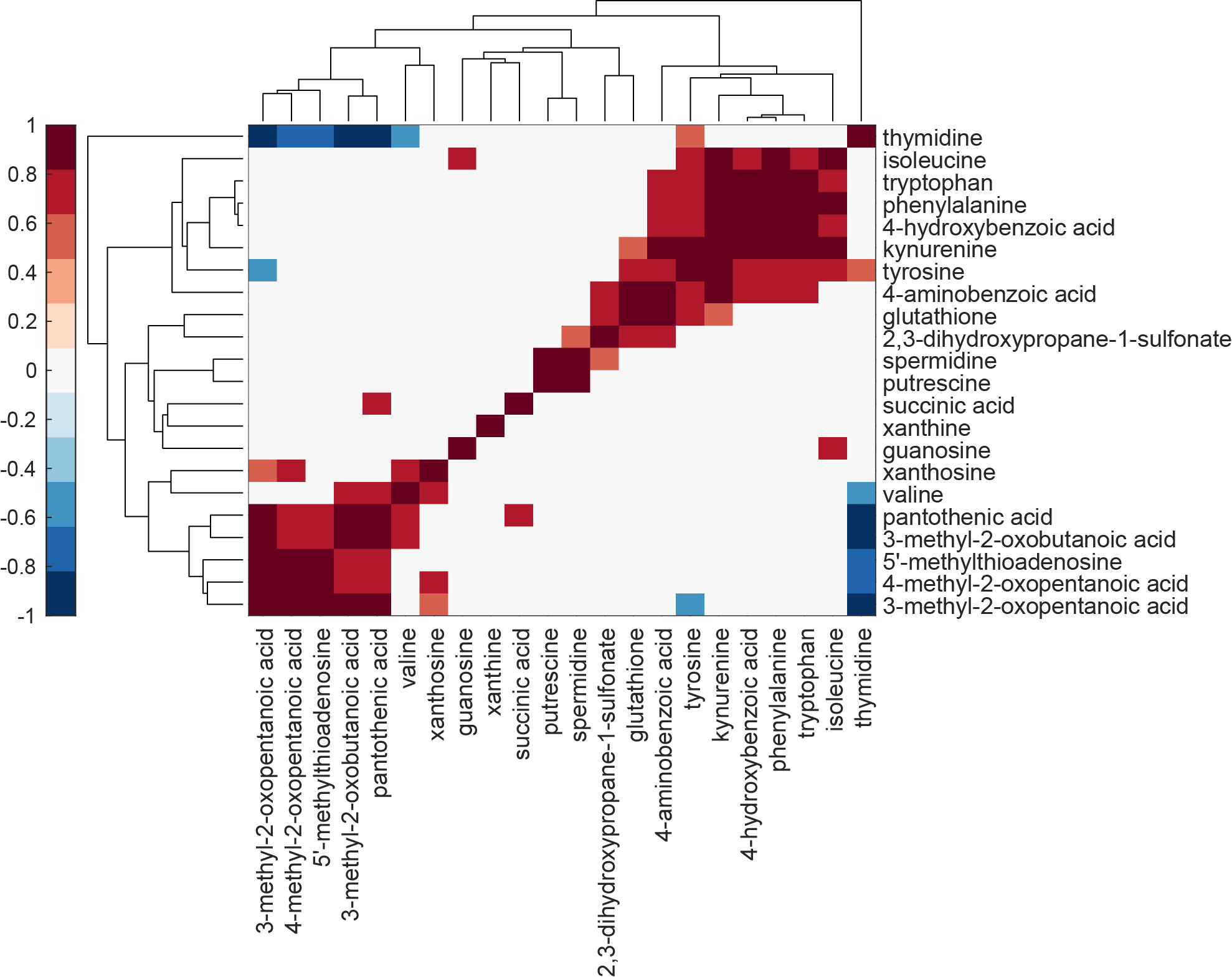
Figure S4.** Clustergram of correlations for all extracellular metabolites collected from cells grown in replete conditions. Statistically significant positive (red) and negative (blue) correlations are Pearson correlations with p-values adjusted using a False Discovery Rate of 5%.

# **References cited**

1. Johnson WM, Kido Soule MC, Kujawinski EB. 2017. Interpreting the impact of matrix on extraction efficiency and instrument response in a targeted metabolomics method. Limnology and Oceanography Methods 15:417-428.

2. Cermak N, Becker JW, Knudsen SM, Chisholm SW, Manalis SR, Polz MF. 2017. Direct single-cell biomass estimates for marine bacteria via Archimedes' principle. ISME J 11:825-828.

3. Atlas RM. 1988. Microbiology. Macmillan Publishing Company, New York, NY.
